# Casparian Strip Fortification as a Defense Mechanism to *Fusarium oxysporum* f. sp. *vasinfectum* Race 4 Infection in a Highly Resistant *Gossypium barbadense* Cultivar

**DOI:** 10.3390/genes16101158

**Published:** 2025-09-29

**Authors:** Stephen Parris, Sonika Kumar, Zhigang Li, Jim Olvey, Mike Olvey, Don C. Jones, Christopher A. Saski

**Affiliations:** 1 Department of Plant and Environmental Sciences, Clemson University, Clemson, SC 29634, USA; sparri2@clemson.edu (S.P.); sonikak@clemson.edu (S.K.); zhiganl@clemson.edu (Z.L.); 2 O&A Enterprises, Maricopa, AZ 85139, USA; oanda999@aol.com (J.O.); mike_naturalhue@yahoo.com (M.O.); 3 Cotton Incorporated, Cary, NC 27513, USA; djones@cottoninc.com

**Keywords:** pima cotton, fusarium wilt race 4, casparian strip, transcriptomics

## Abstract

Background/Objectives: Fusarium wilt of cotton, caused by *Fusarium oxysporum* f. sp. *vasinfectum* (FOV), is a destructive vascular disease that severely impacts cotton production. Among its variants, race 4 (FOV4) is especially aggressive, leading to early season stand losses and yield reductions. While resistant cultivars of *Gossypium barbadense* (pima cotton) have been developed, the molecular basis of this resistance remains unclear. This study aimed to characterize transcriptomic responses associated with FOV4 resistance in pima cotton. Methods: We conducted an in vitro infection assay using two *G. barbadense* cultivars with contrasting phenotypes: the highly resistant ‘DP348RF’ and the highly susceptible ‘GB1031’. Root tissues were sampled at multiple stages of infection, and RNA sequencing was performed to identify differentially expressed genes and pathways contributing to resistance. Results: Resistant plants ‘DP348RF’ showed strong induction of genes related to reactive oxygen species (ROS) metabolism, chitinase activity, and lignification compared to the susceptible cultivar. Notably, genes involved in the biosynthesis and reinforcement of the Casparian strip, a critical biochemical barrier limiting pathogen penetration into vascular tissues, were uniquely and significantly upregulated in resistant roots. These transcriptional responses suggest that fortification of cell wall barriers and enhanced antimicrobial defenses contribute to effective restriction of FOV4 colonization. Conclusions: Our findings identify a distinct molecular signature of resistance to FOV4 in pima cotton, with Casparian strip reinforcement emerging as a potential mechanism limiting vascular infection. These insights provide a foundation for breeding strategies aimed at improving Fusarium wilt resistance in cotton.

## 1. Introduction

Cotton is the world’s leading renewable textile fiber, predominantly derived from two species: *Gossypium hirsutum* (upland) and *G. barbadense* (pima). In the United States, upland cotton accounts for approximately 97% of domestic production, whereas pima cultivars represent the remaining 3% [1]. Pima cotton, also referred to as Egyptian or extra-long-staple (ELS) cotton, is valued for its longer, stronger, and finer fibers which produce softer and higher-quality textiles. Despite these desirable fiber properties, pima cotton is cultivated on a limited scale in the United States, resulting in premium market prices. Its cultivation is constrained by multiple factors including climate, water availability, land-use priorities, economic considerations, market demand, and global competition. In addition, pima cotton generally requires a longer growing season than upland cotton, rendering it more vulnerable to cumulative pest pressures and further limiting its profitable production range [2].

Fusarium wilt, caused by (FOV), is one of the most destructive diseases of both upland and pima cotton, with the potential to devastate entire fields if unmanaged [3,4]. Classification of FOV is based on pathogenicity across cotton species and cultivars, leading to the recognition of seven distinct races: 1, 2, 3, 4, 6, 7, and 8 [5]. More recently, isolates have been characterized genotypically and with vegetative compatibility analysis, identifying more than 23 vegetative compatibility groups (VCG), including two distinct VCGs in Australia [6,7]. Among these, race 4 (FOV4) belonging to VCG0114 and first reported in India in 1960, is especially destructive and highly virulent [5]. FOV4 causes substantial early season stand losses and severe wilting in mature plants often resulting in fatal outcomes and severely reducing production [5]. Its persistence is further exacerbated by the pathogen’s ability to survive for extended periods in soil either in a dormant state as chlamydospores or as a saprophyte on decaying organic matter [8].

FOV4 was first reported in U.S. cotton production in the San Joaquin Valley of California in 2001 [9]. Since its introduction the pathogen has devastated upland cotton in California and has spread eastward into Texas [10]. In response, substantial breeding efforts have focused on developing commercial cultivars with durable resistance to this highly virulent race. Conventional breeding approaches, primarily in the private sector, have been particularly successful in pima cotton, resulting in the release of multiple cultivars that combine FOV4 resistance with high yield potential and superior fiber quality. These advances have had measurable impacts on production trends; for example, USDA NASS reported a 13.7% increase in planted pima acreage in California between 2000 and 2020 [11].

Transcriptome profiling through RNA sequencing (RNA-seq) enables the characterization of gene expression patterns, regulatory networks, and functional pathways within specific cells or tissues. Recent transcriptome and multi-omics studies have shed light on some of the molecular mechanisms used by cotton to resist fungal pathogens like *Fusarium* and *Verticillium dahliae* [12,13,14,15]. These studies have uncovered the roles of specific gene families and metabolic pathways that are the most active in cotton under infection, providing greater understanding of molecular resistance, and targets to enhance resistance in cotton.

In this study we aimed to identify the genes and associated biochemical pathways underlying resistance to FOV4 in pima cotton. To achieve this, we characterized the induced defense response over a time course following infection in a highly resistant and a highly susceptible cultivar. Plants were cultured aseptically and inoculated in a controlled in vitro system designed to isolate host–pathogen interactions and minimize environmental variation. This approach allowed us to uncover resistance-associated responses, including pathways linked to reactive oxygen species, lignification, and Casparian strip fortification, thereby providing new insight into the molecular basis of durable FOV4 resistance in pima cotton [16].

## 2. Materials and Methods

### 2.1. Plant Growth and Inoculations

*G. barbadense* varieties ‘DP348RF’ (resistant) and ‘GB1031’ (susceptible) were provided by James Olvey (O&A Enterprises, Maricopa, AZ 85139, USA). Seeds were germinated and aseptically cultured following the in vitro protocol outlined in [16,17]. FOV4 isolate 89-1A was provided by Dr. Jeff Coleman and originally obtained from Tulare County, California [18]. Isolate 89-1A was obtained under U.S. Department of Agriculture Animal and Plant Health Inspection Service Plant Protection and Quarantine 526 permit (no. P526P-19-02179) issued to Dr. C. Saski. FOV4 cultures were initiated on ½ potato dextrose agar (PDA) and left in the dark to incubate at 28 °C for two weeks. A conidial suspension was prepared using autoclaved, deionized (DI) water and sterile bent rod to dislodge spores, and diluted to a final density of 2.5 × 10^6^ conidia/mL.

Two-week old plants were inoculated by pipetting 200 µL of FOV4 suspension at the base of each plant, and non-inoculated control plants were mock inoculated by pipetting 200 µL of autoclaved DI water at their base. Roots were harvested and flash-frozen in liquid nitrogen for RNA isolation at 12 h, 24 h, and 72 h after inoculation (hai), in addition to 21 days after inoculation to capture both an immediate and latent response to infection. Control roots were harvested 72 h after mock inoculation with sterile, DI water.

### 2.2. RNA Isolation and Sequencing

A total of 3 biological replicates for each timepoint and treatment were collected, with each biological replicate composed of two plants’ roots pooled together and finely ground in the presence of liquid nitrogen for RNA isolation. Total RNA was extracted using a modified CTAB protocol with a lithium chloride precipitation buffer similar to the protocol described in Kumar et al. [19]. mRNA libraries were constructed and sequenced at Novogene, Sacramento, CA. Raw RNA reads were trimmed with Trimmomatic [20] and aligned to the *G. barbadense* ‘3-79’ genome HAU_v1.1 [21] using the bowtie2 short-read aligner [22]. Tissue collected included cells from both the host and fungus, FOV4, read binning was performed to remove transcripts associated with FOV4 instead of the two *G. barbadense* hosts.

### 2.3. Transcritomics and Data Analysis

**Figure 1:**
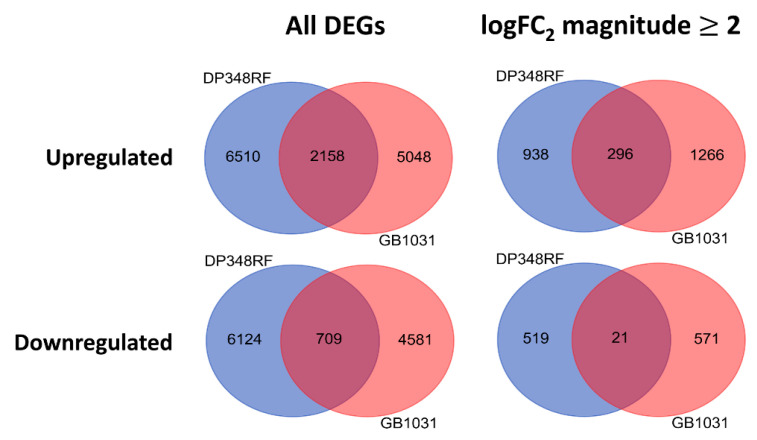
Venn diagram analysis on the abundance and uniqueness of differentially expressed genes (DEGs) in root cells of *G. barbadense* ‘DP348RF’ and ‘GB1031’ 12, 24, and 72 h after inoculation with FOV4.

**Figure 2:**
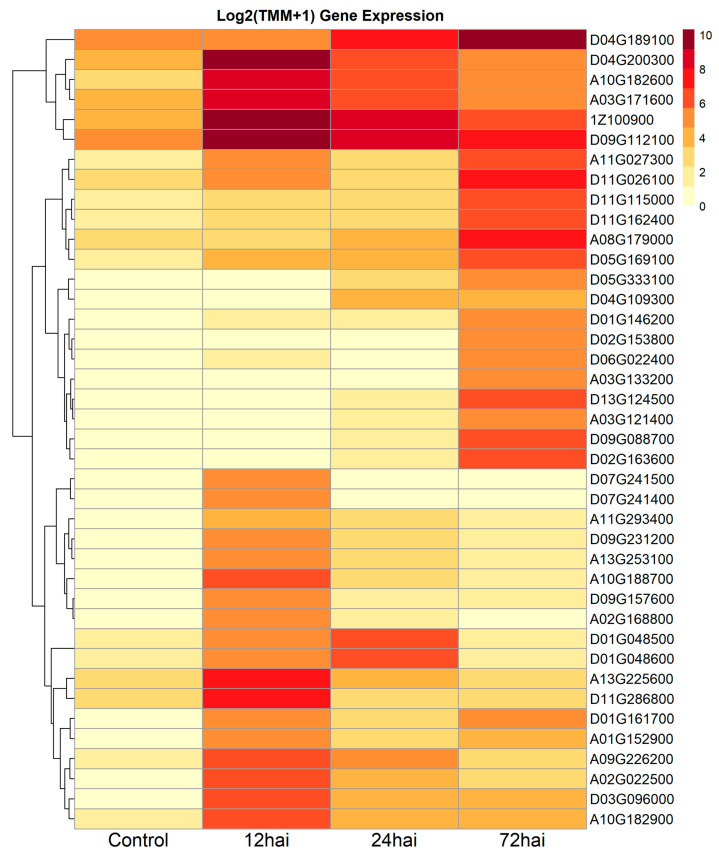
Heat map displaying the Log_2_ adjusted expression values for the top 40 uniquely upregulated genes in *G. barbadense* ‘DP348RF’ root cells in control, 12, 24, and 72 h after inoculation with FOV4.

**Figure 3:**
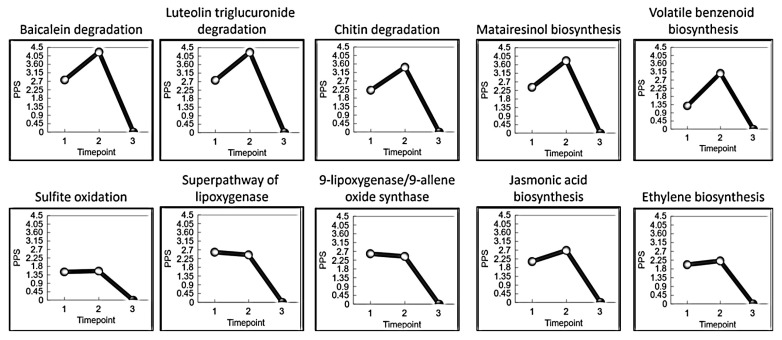
The top ten metabolic pathways with the largest perturbations in *G. barbadense* ‘DP348RF’ root cells after infection with FOV4. Pathway perturbation scores (PPS) measure the magnitude to which a metabolic pathway is up- or down-regulated at each timepoint (1 = 12 h after inoculation (hai), 2 = 24 hai, 3 = 72 hai) compared to the pathway activity in the control samples.

**Figure 4:**
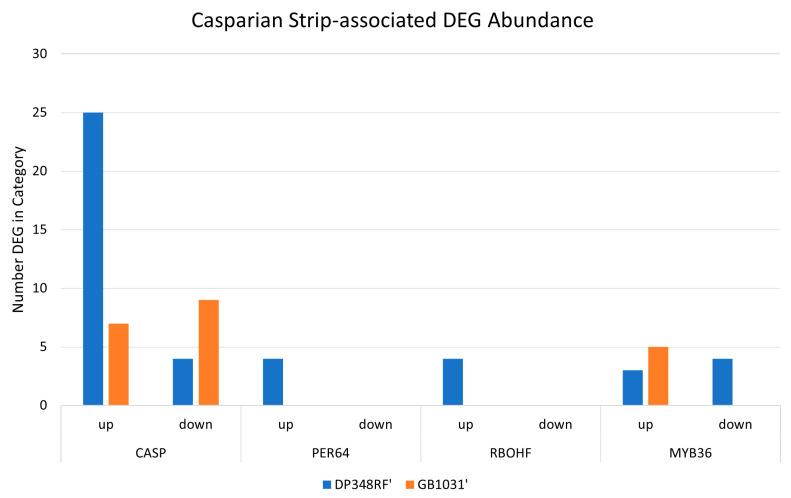
Counts for differentially expressed genes associated with Casparian strip maintenance in *G. barbadense* ‘DP348RF’ and ‘GB1031’ following infection with FOV4. Casparian Strip Membrane Proteins (CASPs), Peroxidase 64 (PER64), Respiratory Burst Oxidase Homolog F (RBOHF) and the transcription factor MYB36.

De novo transcriptomes were analyzed using the Trinity de novo transcriptome pipeline, incorporating edgeR and RSEM for quantification [23,24,25]. Differentially expressed genes (DEGs) were identified by making comparisons between inoculated timepoint and the control samples. To identify resistance-specific responses, we removed DEGs that were also observed in the susceptible ‘GB1031’. Gene ontology enrichment analysis was performed in R using goseq and GO.db packages, expression heat maps were constructed in R using the heatmap.2 function of the gplots package [26,27,28,29]. DEGs were filtered for false discovery rate (FDR) ≤ 0.05, DEGs of any logFC_2_ were considered unless otherwise stated. Metabolomic pathway analysis, via CottonCyc was performed using Pathway Tools software (version 23.0) with MetaCyc database [30,31]. Table 1, Table 2 and Table 3 and Figure 1, Figure 2, Figure 3 and Figure 4 were prepared using the gene expression/RNA-seq data. The source data are provided in Supplemental Tables S1–S3.

## 3. Results

### 3.1. In Vitro FOV4 Infection and Transcriptome Profiling of a Highly Resistant and Susceptible Pima Cultivar

Plants of *G. barbadense* ‘DP348RF’ and ‘GB1031’ were cultured aseptically in the co-culture system described in Parris et al. [16]. At the time of root harvest both genotypes remained asymptomatic; however, the first visible symptoms of Fusarium wilt appeared at 10 days post-inoculation (dpi) in ‘GB1031’ and at 17 dpi in ‘DP348RF’. In the resistant cultivar symptoms developed more slowly and remained relatively mild, with no mortality observed after four weeks in co-culture. In contrast, symptoms in ‘GB1031’ progressed rapidly and with greater severity, ultimately leading to complete plant death within 30 days of inoculation. Disease symptoms in the susceptible cultivar included wilting, defoliation, necrotic foliar lesions, and eventual plant mortality.

In the resistant cultivar ‘DP348RF’ a total of 15,501 genes were differentially expressed (DEGs) in inoculated roots compared to controls, including 1774 genes with an absolute log_2_ fold-change ≥2 (Table S2). In the susceptible cultivar ‘GB1031’ 12,448 DEGs were identified, of which 2154 exhibited an absolute log_2_ fold-change ≥2 (Table S3). Comparative analysis of the DEG sets revealed that the induced genetic response to FOV4 infection in ‘DP348RF’ was largely distinct, sharing only ~25% of upregulated genes and ~10% of downregulated genes with ‘GB1031’ (Figure 1).

Gene expression profiling within the first 72 h post-inoculation with FOV4 revealed the activation of early defense mechanisms in ‘DP348RF’. Among the top 40 uniquely upregulated genes (highest log_2_ fold-change relative to non-inoculated controls), 10 lacked functional annotation, including two of the top five, with the most strongly induced gene, Gobar.D05G333100 (log_2_FC = 5.31), also uncharacterized (Table 1). Annotated genes within this group encoded proteins associated with oxidative stress responses (oxidoreductases, peroxidases), defense-related proteins (dirigent proteins, seed proteins), metabolic enzymes (glucosyltransferases), and regulatory elements (ethylene-responsive transcription factors) (Table 2; Figure 2).

### 3.2. Functional Enrichment Analysis of DEGs in ‘DP348RF’

Functional enrichment analysis of the uniquely upregulated genes in ‘DP348RF’ (log_2_FC ≥ 0; FDR < 0.01) revealed biological processes associated with the resistant defense response to FOV4. Enriched categories included generalized defense responses (GO:0006952), responses to oxidative stress (GO:0006979) and biotic stimuli (GO:0009607), genes related to cell wall organization (GO:0005618) and nucleosome composition (GO:0000786), as well as molecular functions such as hexosyltransferase activity (GO:0016758) and oxidoreductase activity (GO:0016491) (Table 3). Among these, transcription factor activity (GO:0003677) and oxidoreductase activity (GO:0016491) represented the two largest enriched categories following FOV4 infection (Table 3).

Metabolic pathway analysis using the CottonCyc database in CottonGen [32,33] identified pathways with the highest predicted perturbations in infected ‘DP348RF’ roots. These included baicalein degradation (hydrogen peroxide detoxification), luteolin triglucuronide degradation, chitin degradation II, matairesinol biosynthesis, volatile benzenoid biosynthesis I (ester formation), sulfite oxidation IV, the superpathway of lipoxygenase, 9-lipoxygenase and 9-allene oxide synthase, jasmonic acid biosynthesis, and ethylene biosynthesis I (Figure 3). Many of these pathways are linked to oxidative burst signaling, fungal cell wall degradation, and host lignification. Notably, the induction of jasmonic acid and ethylene biosynthetic pathways suggests an important role for hormone-mediated signaling in coordinating the defense response of ‘DP348RF’ to FOV4 infection.

### 3.3. Casparian Strip Biofortification in Response to FOV4 Infection

Analysis of lignification-associated genes revealed a large set of upregulated transcripts linked to Casparian strip formation and maintenance in the ‘DP348RF’ transcriptome (Supplemental Table S1). Key genes involved in Casparian strip development included Casparian strip membrane proteins (CASPs), Peroxidase 64 (PER64), Respiratory Burst Oxidase Homolog F (RBOHF), and the transcription factor MYB36 [34]. Genes from each of these families were significantly upregulated in both ‘GB1031’ and ‘DP348RF’ following infection; however, the response in ‘DP348RF’ was more pronounced, with additional unique sets of induced genes, including 21 CASPs, all 4 PER64 isoforms, and all 4 RBOHF isoforms (Figure 4).

## 4. Discussion

Gene expression studies are powerful tools for identifying candidate genes and biochemical pathways associated with specific traits. They provide high-resolution insights into trait biology, particularly for complex traits influenced by numerous genes and environmental interactions, such as pathogen resistance. In this study, we characterized transcriptomic changes in pima cotton root cells following FOV4 infection to investigate the molecular mechanisms underlying the heritable resistance observed in *G. barbadense* ‘DP348RF’.

We observed a rapid induction of host defense genes as early as 12 h after inoculation, highlighting the importance of early transcriptional reprogramming in resistance to FOV4. A substantial fraction of highly upregulated genes (absolute log_2_FC ≥ 2, FDR < 0.01) lacked functional annotation, underscoring the need for species-specific functional validation to fully characterize the molecular basis of resistance in pima cotton. Among the annotated genes strongly induced within the first three days of infection in the resistant cultivar, we identified enrichment of categories commonly associated with biotic defense responses, including oxidoreductases, peroxidases, seed proteins, dirigent proteins, glucosyltransferases, and ethylene-responsive transcription factors.

Oxidoreductases and peroxidases are well-established components of early defense against fungal pathogens. They contribute to lignin and suberin biosynthesis, cell wall cross-linking, phytoalexin production, and the metabolism of reactive oxygen species (ROS) [35]. In ‘DP348RF’ these genes were significantly upregulated within 12 h post-infection and remained elevated through 72 h, consistent with an oxidative burst response. Sustained expression of these enzymes may reinforce cell walls via lignin and suberin deposition around infection sites, restricting fungal colonization [36]. The enriched ROS signature likely serves dual functions: generating antimicrobial molecules that directly inhibit fungal growth, and strengthening cellular structures that act as barriers to invasion. Importantly, this oxidative activity aligns with the enhanced expression of Casparian strip–associated genes in ‘DP348RF’, suggesting that ROS-mediated lignification contributes directly to fortification of this barrier and may limit vascular colonization by FOV4.

In addition to oxidoreductases and peroxidases, we observed significant upregulation of chitinases in infected ‘DP348RF’ roots, with 15 genes exhibiting an absolute log_2_FC ≥ 2 (Supplemental Table S2). Chitin hydrolysis not only compromises fungal cell walls but also generates fragments that act as elicitors of pathogen-associated molecular pattern (PAMP) signaling, amplifying innate immunity through positive feedback [37,38,39,40]. Chitinase activity has also been shown to enhance ROS production during fungal infection [37]. The co-upregulation of chitinases with oxidoreductases and peroxidases suggests a coordinated defense in which chitin degradation, ROS accumulation, and phytoalexin production act synergistically to restrict FOV4 colonization.

The CottonCyc database, curated by CottonGen, integrates experimentally observed metabolic pathways with cotton transcriptomes using the Pathway Tools platform [32,33,41]. In the absence of direct metabolic profiling, this resource enables prediction of metabolic perturbations based on transcriptomic data by mapping gene products to reactions and pathways. Using this approach, we identified several pathways strongly perturbed in ‘DP348RF’ roots following FOV4 infection, including: baicalein degradation (hydrogen peroxide detoxification), chitin degradation II, matairesinol biosynthesis (lignan synthesis), jasmonic acid biosynthesis, ethylene biosynthesis, and lipoxygenase-associated pathways (Figure 3). These pathways align with well-established defense signatures and can be grouped into three major categories: ROS-associated processes, phytoalexin production, and hormone-mediated defense signaling.

**Figure 5:**
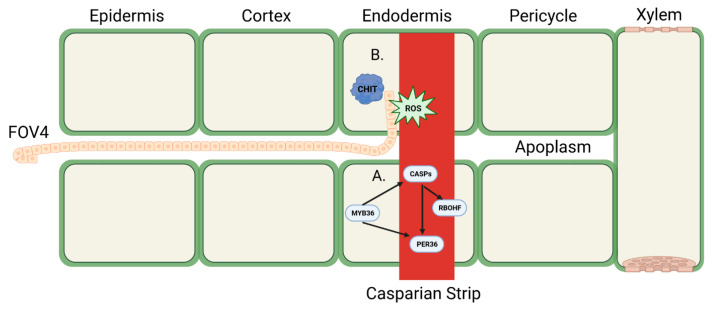
Potential role of the Casparian strip in the restriction of vascular colonization by FOV4. (**A**) Upregulation of genes involved in Casparian strip fortification following infection. (**B**) Subsequent induction of Chitinase (CHIT) and reactive oxygen species (ROS) may act to further weaken the invading pathogen. Created in BioRender. Parris, S. (2025) https://BioRender.com/sycgihc.

Analysis of these pathways and genes suggests that ‘DP348RF’ mounts a coordinated defense response centered on enhanced tissue lignification, particularly in the root endodermis at the Casparian strip. The Casparian strip is a lignin-rich modification in endodermal cells regulates the solute movement into the vascular system [42,43]. By blocking apoplastic flow and forcing solutes to pass through endodermal cells, it plays a critical role in selective nutrient and water uptake. More recently, it has also been implicated in restricting vascular pathogens such as VERTDA and its secreted effectors [44,45]. In our study, genes central to Casparian strip formation and reinforcement, including *CASP*, *PER64*, *RBOHF*, *MYB36*, and *PR1*, were significantly upregulated in ‘DP348RF’ following FOV4 infection, supporting the hypothesis that fortification of this barrier contributes to resistance. A strengthened Casparian strip may slow fungal progression toward the vasculature by diverting growth into intracellular spaces where plants deploy a wider arsenal of defenses, including oxidative bursts and phytoalexin activity (Figure 5).

While most of the significant differentially expressed genes in the *G. barbadense* transcriptome have functional annotations, many highly expressed genes in ‘DP348RF’ have yet to be annotated. To understand the roles of these genes in the induced pathogen defense response further investigation is necessary. Direct functional studies including knockdowns or overexpression of these unannotated genes will be essential to further understanding of the resistance to FOV4 deployed by pima cotton.

Taken together, our findings suggest that resistance to FOV4 in *G. barbadense* ‘DP348RF’ likely involves a two-tiered defense strategy. First, we note a rapid induction of Casparian strip biosynthetic genes potentially leading to the reinforcement of a physical and biochemical barrier at the root endodermis. This reinforcement can restrict pathogen entry into the vascular system through cell membranes rather than via the apoplast [46,47,48,49]. Second, this barrier forces the fungus into intracellular compartments, where host defenses, including reactive oxygen species and phytoalexin production, such as chitinase activity, further degrade fungal structures and restrict colonization. This integrated strategy highlights the role of Casparian strip fortification as a key component of resistance in pima cotton and demonstrates how structural barriers and inducible biochemical defenses can act synergistically to suppress FOV4 infection.

## Figures and Tables

**Table 1:** Summary of Differentially Expressed Genes (DEGs) in root cells of *G. barbadense* ‘DP348RF’ and ‘GB1031’ 12, 24, and 72 h after inoculation with FOV4. Only DEGs with a false discovery rate *p*-value < 0.01 were considered.

	All DEGs					
	All		Up		Down	
	total	unique	total	unique	total	unique
Resistant (DP348RF)	15,501	9984	8668	6510	6833	6124
Susceptible (GB1031)	12,448	6931	7206	5048	5290	4581
Shared	5517		2158		709	
	**DEGs LogFC_2_** ≥ **2**					
	All		Up		Down	
	total	unique	total	unique	total	unique
Resistant (DP348RF)	1774	1261	1234	938	540	519
Susceptible (GB1031)	2154	1641	1562	1266	592	571
Shared	513		296		21	

**Table 2:** Top upregulated genes containing annotations in *Gossypium barbadense* ‘DP348RF’ after inoculation with FOV4. Comparisons were made between inoculated timepoints and non-inoculated control samples.

Gene	logFC	FDR	Best Hit *Arabidopsis*	Gene Defline
Gobar.A10G188700.1	5.21	6.11 × 10^−44^	AT2G36780.1	GLUCOSYL/GLUCURONOSYL TRANSFERASES//UDP-GLYCOSYLTRANSFERASE 73C7
Gobar.A11G027300.1	5.09	6.39 × 10^−9^	AT4G12520.1	Protease inhibitor/seed storage/LTP family (Tryp_alpha_amyl)
Gobar.D01G161700.1	4.93	4.65 × 10^−8^	AT2G37870.1	(FAMILY NOT NAMED//NON-SPECIFIC LIPID-TRANSFER PROTEIN)
Gobar.A02G022500.1	4.8	2.64 × 10^−23^	AT4G14040.1	56kDa selenium binding protein (SBP56) (SBP56)
Gobar.A13G253100.1	4.73	5.13 × 10^−7^	AT1G08080.1	CARBONIC ANHYDRASE//ALPHA CARBONIC ANHYDRASE 3
Gobar.D07G241500.1	4.69	6.83 × 10^−38^	AT3G23230.1	ETHYLENE-RESPONSIVE TRANSCRIPTION FACTOR ERF098
Gobar.D01G048500.1	4.63	5.30 × 10^−24^	AT4G38700.1	NUCLEOPORIN-RELATED//DIRIGENT PROTEIN 15-RELATED
Gobar.D01G048600.1	4.63	5.30 × 10^−24^	AT4G38700.1	NUCLEOPORIN-RELATED//DIRIGENT PROTEIN 15-RELATED
Gobar.D06G022400.1	4.62	7.89 × 10^−5^	AT4G34150.1	SYNAPTOTAGMIN
Gobar.D07G241400.1	4.6	1.70 × 10^−5^	AT3G23230.1	ETHYLENE-RESPONSIVE TRANSCRIPTION FACTOR ERF098
Gobar.A01G152900.1	4.5	5.02 × 10^−15^	AT2G37870.1	FAMILY NOT NAMED//NON-SPECIFIC LIPID-TRANSFER PROTEIN
Gobar.D11G026100.1	4.48	5.75 × 10^−13^	AT4G12480.1	Hydrophobic seed protein (Hydrophob_seed)
Gobar.D05G169100.1	4.39	7.44 × 10^−18^	AT2G33790.1	ARABINOGALACTAN PROTEIN 31
Gobar.D11G286800.1	4.39	6.85 × 10^−41^	AT1G60690.1	Perakine reductase
Gobar.A12G087900.1	4.36	2.08 × 10^−26^	AT4G37980.1	cinnamyl-alcohol dehydrogenase (E1.1.1.195)
Gobar.D03G195800.1	4.34	2.87 × 10^−5^	AT4G17030.1	EXPANSIN-LIKE B1
Gobar.A05G123700.1	4.31	1.91 × 10^−6^	AT1G76690.1	NADH OXIDOREDUCTASE-RELATED
Gobar.A06G015300.1	4.22	6.97 × 10^−12^	AT4G31940.1	Cytochrome P450 CYP2 subfamily
Gobar.A09G113600.1	4.18	3.79 × 10^−19^	AT5G60520.1	Root cap (Root_cap)
Gobar.D02G024900.1	4.18	1.99 × 10^−12^	AT1G05530.1	anthocyanidin 3-O-glucoside 5-O-glucosyltransferase (UGT75C1)
Gobar.D12G083200.1	4.18	9.43 × 10^−9^	AT4G37990.1	cinnamyl-alcohol dehydrogenase (E1.1.1.195)
Gobar.A13G225500.1	4.17	4.07 × 10^−13^	AT4G03070.1	OXIDOREDUCTASE, 2OG-FE II OXYGENASE FAMILY PROTEIN
Gobar.A04G009300.1	4.15	3.96 × 10^−6^	AT2G21110.1	NUCLEOPORIN-RELATED//DIRIGENT PROTEIN 15-RELATED
Gobar.D06G159700.1	4.14	8.78 × 10^−29^	AT5G45890.1	Cysteine proteinase Cathepsin F//Cysteine proteinase Cathepsin L
Gobar.A12G058100.1	4.04	1.28 × 10^−12^	AT4G37370.1	E1.14.-.-
Gobar.D01G048400.1	4.03	6.41 × 10^−6^	AT4G38700.1	NUCLEOPORIN-RELATED//DIRIGENT PROTEIN 15-RELATED
Gobar.D13G269500.1	4.02	2.97 × 10^−8^	AT1G08080.1	CARBONIC ANHYDRASE//ALPHA CARBONIC ANHYDRASE 3
Gobar.A05G421900.1	3.99	2.92 × 10^−28^	AT5G05340.1	PEROXIDASE 52
Gobar.D12G160200.1	3.97	6.74 × 10^−15^	AT1G03870.1	FAMILY NOT NAMED//FASCICLIN-LIKE ARABINOGALACTAN PROTEIN 13-RELATED

**Table 3:** The top five enriched gene ontologies in *G. barbadense* ‘DP348RF’ root cells under infection by FOV4 for each of the three categories: biological processes (BP), cellular components (CC), and molecular functions (MF).

Ontology	Over Represented *p*-Value	Associated DEGs	Total Genes with Ontology	Term	Category
GO:0008152	1.49 × 10^−10^	48	202	metabolic process	BP
GO:0006979	8.05 × 10^−6^	22	82	response to oxidative stress	BP
GO:0009607	1.31 × 10^−5^	12	30	response to biotic stimulus	BP
GO:0006952	1.81 × 10^−5^	14	41	defense response	BP
GO:0007017	3.82 × 10^−5^	8	16	microtubule-based process	BP
GO:0000786	3.17 × 10^−12^	16	22	nucleosome	CC
GO:0005874	4.67 × 10^−6^	9	19	microtubule	CC
GO:0005840	0.000143471	27	130	ribosome	CC
GO:0005622	0.000568915	23	115	intracellular anatomical structure	CC
GO:0005618	0.00094564	10	34	cell wall	CC
GO:0016758	4.05 × 10^−13^	28	63	hexosyltransferase activity	MF
GO:0003677	6.44 × 10^−12^	58	266	DNA binding	MF
GO:0020037	4.59 × 10^−1^	54	223	heme binding	MF
GO:0016491	4.15 × 10^−9^	56	275	oxidoreductase activity	MF
GO:0009055	6.95 × 10^−9^	23	63	electron transfer activity	MF

## Data Availability

Adaptor-trimmed sequences have been uploaded to NCBI SRA and are available under accession PRJNA1332781.

## Supplementary Materials

The following supporting information can be downloaded at: https://www.mdpi.com/article/10.3390/genes16101158/s1, Table S1: Differentially expressed genes associated with Casparian strip formation; Table S2: Differentially expressed genes in *G. barbadense* ‘DP348RF’; Table S3: Differentially expressed genes in *G. barbadense* ‘GB1031’; Table S4: top 40 upregulated genes in ‘DP348RF’ including genes without annotation; Table S5: Number of DEGs in CottonCyc predicted pathways; Table S6: RNAseq alignment statistics.

## Abbreviations

The following abbreviations are used in this manuscript:

FOV4	*Fusarium oxysporum* f. sp. *vasinfectum* race 4
ROS	Reactive oxygen species
ELS	Extra-long staple
VCG	Vegetative compatibility groups
PDA	Potato dextrose agar
DI	Deionized
hai	Hours after inoculation
DEG	Differentially expressed gene
dpi	Days post inoculation
GO	Gene ontology
BP	Biological processes
CC	Cellular components
MF	Molecular functions
PPS	Pathway perturbation scores
CASP	Casparian strip membrane protein
PER64	Peroxidase 64
RBOHF	Respiratory burst oxidase homolog F
PAMP	Pathogen-associated molecular pattern
